# Evaluation of a Novel Multiplex High-Definition PCR Assay for Detection of Tick-Borne Pathogens in Whole-Blood Specimens

**DOI:** 10.1128/JCM.00513-19

**Published:** 2019-10-23

**Authors:** Blake W. Buchan, Dean A. Jobe, Michael Mashock, Derek Gerstbrein, Matthew L. Faron, Nathan A. Ledeboer, Steven M. Callister

**Affiliations:** a Department of Pathology, The Medical College of Wisconsin, Milwaukee, Wisconsin, USA; b Microbiology Research and Molecular Diagnostics Laboratories, Gundersen Medical Foundation, La Crosse, Wisconsin, USA; University of Tennessee at Knoxville

**Keywords:** high-definition PCR, tick-borne pathogens

## Abstract

The prevalence of tick-borne infections has been steadily increasing in both number and geographic distribution in the United States and abroad. This increase, in conjunction with the continued recognition of novel pathogens transmitted by ticks, has made accurate diagnosis of these infections challenging. Mainstay serologic tests are insensitive during the acute phase of infection and are often cross-reactive with similar pathogenic and nonpathogenic organisms. Further, they are unable to reliably differentiate active versus past infection which can lead to misdiagnosis and incorrect understanding of the epidemiology and incidence of specific tick-borne pathogens. We evaluated a novel multiplexed high-definition PCR (HDPCR) Tickborne Panel (TBP) assay (ChromaCode, Carlsbad, CA) for the detection of nine tick-borne pathogens or groups associated with human illness. The HDPCR technology enables multiplex identification of multiple targets in a single fluorometric channel based on fluorescent signal modulation using a limiting probe design. A collection of 530 whole-blood specimens collected from patients being evaluated for tick-borne infections, in addition to a panel of 93 simulated specimens, were used to challenge the HDPCR TBP. The results were compared to a clinically validated traditional multiplexed PCR test with additional sequence analysis and clinical history collected to aid in resolving discrepancies. Among clinical specimens the TBP demonstrated 100% sensitivity for the identification of Anaplasma phagocytophilum, Borrelia miyamotoi, Borrelia mayonii, and Rickettsia rickettsii. The sensitivity for identification of B. burgdorferi was 44.4% compared to a composite gold standard. Among simulated specimens containing single or multiple targets present at 10^3^ to 10^5^ copies/PCR, the sensitivity of TBP was 100% for all targets, with a combined specificity of 99.5%. Of note, an increased rate of false-positive results was observed among simulated specimens that contained multiple targets. Based on these data, we find the HDPCR TBP to be a useful adjunct for the diagnosis of tick-borne infections in patients with suspected tick-borne illness.

## Abstract (precis)

The prevalence of tick-borne infections has been steadily increasing in both number and geographic distribution in the United States and abroad. This increase, in conjunction with the continued recognition of novel pathogens transmitted by ticks, has made accurate diagnosis of these infections challenging. Mainstay serologic tests are insensitive during the acute phase of infection and are often cross-reactive with similar pathogenic and nonpathogenic organisms.

## INTRODUCTION

Tick-borne infections, especially those transmitted by *Ixodes* spp., are increasing in prevalence in many regions of the United States. The number of reported cases of tick-borne illness has doubled during a 10-year interval, reaching approximately 50,000 in 2016 (1). However, when the number of unreported cases is included the actual number of tick-borne infections may approach 500,000 annually (2, 3). Approximately 75% of cases with an identified etiology are attributable to *Borrelia* spp. that cause Lyme disease, but infections due to other recognized and novel tick-borne pathogens, including Anaplasma phagocytophilum, *Ehrlichia* spp., *Rickettsia* spp., *Babesia* spp., and relapsing fever *Borrelia* spp., are also increasing (1, 4–7). For example, two novel *Borrelia* spp. transmitted by *Ixodes* ticks, *B. mayonii* and B. miyamotoi, were only recently recognized as agents of tick-borne illness in the United States (8, 9). While *B. mayonii* is closely related to B. burgdorferi and causes a Lyme disease-like illness, B. miyamotoi is a genetically distinct relapsing fever group *Borrelia* spp. associated with a different clinical course (8, 10).

The rise in reported cases of tick-borne illness is likely multifactorial, involving the dispersal of tick vectors into new geographic regions, increased awareness among clinicians, improved diagnostic approaches, and the ongoing recognition of new pathogens transmitted by tick bite. Increased likelihood of contracting multiple tick-borne infections, both concurrently and longitudinally, has further complicated accurate laboratory diagnosis. For example, widely used serologic methods for confirming Lyme disease, anaplasmosis, and ehrlichiosis suffer from low sensitivity during the acute phase of infection (4, 11, 12) and poor specificity due to cross-reactive antibodies (8, 10, 13). Further, serologic tests are often incapable of differentiating current from past infection. These factors all contribute to the potential for incorrect diagnosis and underreporting of these infections. Alternatively, direct microscopic examination of blood smears can be helpful for confirmation of some tick-borne pathogens that achieve a high concentration in blood, but the procedure can be tedious, insensitive, and subjective and may often provide false-negative results.

PCR-based approaches for the diagnosis of tick-borne infections are gaining acceptance and are currently recommended for the diagnosis of several tick-borne pathogens (4, 6, 7, 14). Specific advantages of PCR-based methods include direct confirmation of infection, the potential for multiplexing to detect multiple organisms in a single assay, and increased sensitivity during the acute phase of infection prior to development of an antibody response. The ability to detect small numbers of organisms can also extend the diagnostic window for up to 30 days for A. phagocytophilum and *Ehrlichia* spp. compared to microscopic examination of blood smears (12). Despite these advantages, molecular diagnostics for tick-borne pathogens remain largely restricted to specialized reference laboratories. These tests differ in the specific organisms detected and are not comprehensive. A 2018 report to congress by the Tickborne Disease Working Group called for the development and evaluation of new technologies for the diagnosis of Lyme disease and other tick-borne illnesses, including molecular and multiplexed approaches (https://www.hhs.gov/sites/default/files/tbdwg-report-to-congress-2018.pdf).

**FIG 1:**
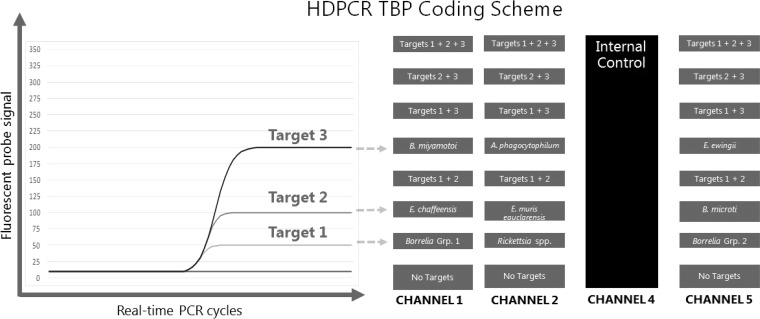
HDPCR uses a different probe concentration for each target in a single fluorescent channel to achieve unique amplification curves for each target. A novel mathematical algorithm was used to analyze amplification curves and detect and differentiate up to six unique targets in a single fluorescent channel. The presence of multiple targets in a single channel results in an additive fluorescence effect that can be differentiated from single-target amplification curves.

The research-use-only (RUO) high-definition PCR (HDPCR) Tickborne Panel (TBP; ChromaCode, Carlsbad, CA) is a multiplex molecular assay that detects and differentiates nine distinct pathogens or pathogen groups associated with tick-borne illness. Individual results are reported for Anaplasma phagocytophilum, Borrelia miyamotoi, *Borrelia* group 1 (B. burgdorferi and *B. mayonii*), *Borrelia* group 2 (B. hermsii, *B. parkeri*, and *B. turicate*), Ehrlichia chaffeensis, Ehrlichia ewingii, Ehrlichia muris subsp. *eauclarensis*, *Rickettsia* spp., and Babesia microti. HDPCR utilizes standard real-time PCR (RT-PCR) and well-established fluorescently labeled hydrolysis probe (TaqMan) chemistry. Unique primer and probe sequences are used for each of the nine assay targets (Table 1); however, multiple unique probes share a fluorophore and are therefore detected in the same fluorometric channel by the RT-PCR instrument. Target differentiation within a single channel is achieved by varying the probe concentration for each target in a given channel. This probe-limiting design results in differences in endpoint fluorescent signal, i.e., maximal PCR curve amplitude or plateau, that are characteristic for each target (Fig. 1). Multiple targets in a single channel result in an additive effect on the maximal fluorescence signal (curve amplitude), which can be predicted based on the concentration of probe used for each individual target. A mathematical algorithm is used to analyze raw RT-PCR amplification data (PCR curves) to identify and differentiate up to six unique targets per real-time PCR fluorometric channel. This HDPCR approach has previously been applied to successfully identify nine viral agents in simulated nasopharyngeal specimens across a wide concentration of 10^1^ to 10^5^ genomic copies/PCR (15). Single channel multiplexing allows expansion of multiplex capabilities enabling identification of the nine TBP assay targets and an internal control using just four channels of a standard real-time PCR instrument (Fig. 1).

**TABLE 1:** Specific genes targeted by the HDPCR TBP assay

Species or group	Gene target	Gene ID^*a*^	Gene full name
*A. phagocytophilum*	APH_RS04060	3930425	RNA polymerase subunit beta (*rpoB*); *A. phagocytophilum* strain HZ
*Borrelia* group 1^*b*^ (*B. burgdorferi* and *B. mayonii*)	*ospA*	1194357	Outer surface protein A; *B. burgdorferi* B31
*Borrelia* group 2 (*B. hermsii*, *B. parkeri*, and *B. turicatae*)	BH0214B	6276532	Glycerophosphodiester phosphodiesterase; *B. hermsii* DAH
*B. miyamotoi*	*glpQ*	35888597	Glycerophosphodiester phosphodiesterase; *B. miyamotoi* LB-2001
*E. chaffeensis*	120 kDa	NA^*c*^	120-kDa outer membrane protein
*E. ewingii*	P28	NA	28-kDa outer membrane protein
*E. muris* subsp. *eauclarensis*	P28-14	NA	Outer membrane protein P28-14
*Rickettsia* spp.	MC1_07110	11994841	17-kDa surface antigen; *R. parkeri* strain Portsmouth
*B. microti*	*cox1*	29141170	Cytochrome oxidase subunit 1; *B. microti*

a NCBI gene ID.

b Assay primers and probes are specific to B. burgdorferi and *B. mayonii*. However, cross-reactivity may be observed with *B. afzellii* if present at concentrations of 10^6^ copies/PCR or higher due to sequence similarity in the genetic target targeted by TBP.

c NA, not applicable. The gene ID is not in the NCBI database. Targets were based on a literature search to identify genes successfully used with real-time PCR for each organism. BLASTn nucleotide BLAST was used to further evaluate potential gene targets. Geneious was used to create alignments and for primer/probe designs. NCBI Primer-BLAST was used for additional bioinformatic inclusivity/exclusivity analysis.

The primary aim of this study was to provide a preliminary evaluation of the performance of the TBP assay and HDPCR technology for the identification of tick-borne pathogens in human and simulated specimens. Residual blood samples collected from patients being evaluated for possible tick-borne illness contracted in the upper Midwest were tested using the HDPCR TBP. TBP results were compared to results obtained using a traditional multiplex real-time PCR test currently used for diagnosis of tick-borne infections at the Gundersen Health System (GHS) (16–18). A panel of simulated specimens were used to evaluate the performance of TBP for rare targets not found during prospective enrollment and to evaluate the ability of the HDPCR technology to detect multiple pathogens in a single specimen. Our findings confirmed that the HDPCR TBP accurately detected and differentiated nine tick-borne pathogens in the simulated samples, and the test yielded results that closely matched the clinical findings obtained using a traditional multiplex PCR test. Therefore, the HDPCR could be considered as an adjunct for confirming tick-borne infection in symptomatic patients.

## MATERIALS AND METHODS

### Patient blood sample enrollment.

This study included 530 residual whole blood samples collected in purple-top (EDTA) vacutainer tubes at GHS, La Crosse, WI, and The Medical College of Wisconsin (MCW), Milwaukee, WI. A total of 450 samples were obtained between 1 June and 15 October 2018 (MCW, *n* = 175; Gunderson Medical Foundation [GMF], *n* = 250) from patients with suspected tick-borne illness. Specifically, blood specimens submitted for clinical testing at GMF during the month of June were included, and specimens with >1 ml of residual whole blood obtained from patients with a clinical order for Lyme serologic test at MCW between 18 June and 15 October 2018 were included. An additional 80 whole-blood specimens were obtained from patients who presented with clinical abnormalities not associated with tick-borne infection to serve as presumed negative clinical specimens (MCW, *n* = 40; GHS, *n* = 40).

### HDPCR TBP.

Testing was conducted at MCW using the commercially available, the RUO HDPCR TBP assay (ChromaCode, Carlsbad, CA) according to the manufacturer’s instructions (v1.0). These data are available elsewhere (https://chromacode.com/news-releases/white-paper-highlights-design-and-analytical-performance-of-the-hdpcr-tick-borne-pathogen-tbp-panel-ruo/). Nucleic acid was extracted from 200 μl of whole blood using a DNA blood minikit (Qiagen) at the GMF or the automated eMAG instrument (bioMérieux) at MCW and eluted into a final volume of 50 μl. DNA concentration in extracts was not determined. All specimens enrolled at GMF were extracted at the time of collection, and extracts were stored at −70 to –80°C for up to 4 months prior to shipping (overnight on dry ice) to MCW for TBP testing. Residual whole blood specimens enrolled at MCW were stored at −70 to –80°C for up to 4 months prior to nucleic acid extraction. These extracts were stored at −70 to –80°C for up to 4 weeks prior to batch TBP testing at MCW and shipment to GMF for reference testing. After TBP testing, the remaining nucleic acid extract volume was refrozen and, in instances of discordant results, was sent back to GMF or MCW (depending on point of origin) for repeat testing by reference PCR and TBP to determine whether the freezing and storage of extracts had impacted specimen integrity.

The TBP assay consist of a frozen ready to use “master mix” that contains nine unique primer sets (forward and reverse) and nine unique TaqMan hydrolysis probes designed to target conserved regions of genes corresponding to each TBP target (Table 1). Specific primer sequences used in the TBP assay are proprietary and cannot be included in the published manuscript. Readers may contact ChromaCode for more information on specific primer sequences. Each unique TaqMan probe is conjugated to one of four fluorophores: FAM (*Borrelia* group 1, *E. chaffeensis*, and B. miyamotoi), ATTO 532 (A. phagocytophilum, *E. muris* subsp*. eauclarensis*, and *Rickettsia* spp.), ATTO 647 (*Borrelia* group 2, *E. ewingii*, and B. microti), and ROX (internal control [IC]). The IC is an MS2 bacteriophage sequence that has been cloned into a circular plasmid. The IC is designed to be added to 200 μl of the whole-blood specimen prior to extraction at a final concentration of 10,000 copies/200 μl to achieve a final concentration of ∼10^3^ copies of IC/PCR. However, the IC was not available at the time of extraction for specimens enrolled at GMF. For these specimens, a volume of the IC equivalent to ∼10^3^ total copies was added to the final PCR (i.e., after nucleic acid extraction). While this does not control for extraction efficiency, it does provide a control for the presence of PCR inhibitors. Since both TBP and a reference PCR were conducted postextraction on a nucleic acid template, the method comparison is valid, provided the IC for both assays was detected.

To initiate TBP testing, nucleic acid extracts were thawed, briefly vortexed, and centrifuged prior to setting up individual PCRs. Each reaction consisted of 5 μl of extracted nucleic acid and 15 μl of TBP master mix in individual wells of a MicroAmp optical 96-well reaction plate (Thermo Fisher Scientific). Four manufacturer-provided calibrators were included with each RT-PCR plate run. Each calibrator consists of a synthetic double-stranded DNA template, along with complementary primer/probe sets titrated to provide an amplification signal at each of the four levels corresponding to single-target and IC detections (see Fig. 1). Inclusion of the calibrators is necessary to define the different fluorescence intensity levels corresponding to the variable probe concentrations in order to ensure proper interpretation and accuracy of results. RT-PCR was conducted using an Applied Biosystems (ABI) 7500 Fast DX instrument. Thermocycling and fluorescence detection parameters were programmed in accordance with the HDPCR TBP instructions for use (v1.0).

Upon completion of RT-PCR, raw data files were exported to a local computer with internet access and were uploaded to the ChromaCode Cloud for analysis. The ChromaCode Cloud is a cloud-based program that applies a proprietary mathematical algorithm to analyze amplification and calibrator data (PCR curves and maximum fluorescence signal) in each fluorescence channel. Raw data are analyzed within 2 min of upload, and a final report of positive for a specific target, negative, or invalid result for each well is generated. For laboratories that are unable to access or utilize cloud-based applications, the ChromaCode Cloud analysis program can alternatively be loaded onto a resident computer for on-site data analyses.

Technologists and principal investigators were blinded to the reference PCR results until all results were reported. Cloud-based analysis of HDPCR run data are automated; however, ChromaCode employees were also blinded to reference PCR results until analyses were completed.

### Reference PCR method.

Reference testing (REF) of nucleic acid extracts was conducted at the GMF using a laboratory-developed multiplex real-time PCR test used to routinely evaluate GHS patients for tick-borne illness. The test simultaneously targets a unique species-specific region of *msp2* of A. phagocytophilum (16, 18), a genus-specific region of the 23s rRNA gene of *Borrelia* spp. (16), and a species-specific region of the 18s rRNA gene of Babesia microti (17). A 5-μl volume of extracted DNA was combined with 20 μl of a master mix that contained 12.5 μl of buffer (10× AmpliTaq Gold buffer, 2.5 mM MgCl_2_, and deoxynucleoside triphosphates), 4.5 μl of a primer/probe mix containing ApMSP2 forward (5′-ATGGAAGGTAGTGTTGGTTATGGTATT-3′), ApMSP2 reverse (5′-TTGGTCTTGAAGCGCTCGTA-3′), ApMSP2 probe (5′-6-carboxyfluorescein [FAM]-TGGTGCCAGGGTTGAGCTTGAGATTG-[BHQ1a]-3′), Bb23S forward (5′-CGAGTCTTAAAAGGGCGATTTAGT-3′), Bb23S reverse (5′-TTATGAAAAAATATTTATTGGGAAT-3′), Bb23S probe (5′-6-ROX-AGATGTGGTAGACCCGAAGCCGAGTG-[BQH-2]-3′), Bab forward (5′-TCGCGTGGCGTTTATTAGAC-3′), Bab reverse (5′-CCGGCAAAGCCATGCGATT-3′), and Bab probe (5′-CY5-AACCAACCCTTCGGGTAATCGGTG[BHQ2]-3′), as well as 2.5 μl of exogenous sample processing control primer and probe containing forward primer (5′-CCTGTGCGGGCAAGAAAG-3′), reverse primer (5′-CGCATCCAGTGCGAAGGT-3′), and probe (5′-HEX-CGAGTTTAACGACAAGCCCAAAGTCA-[BHQ1a]-5HEX-3′), and 0.5 μl of AmpliTaq Gold DNA polymerase (1.5 U; Life Technologies). Real-time PCR was conducted using a model 3000P thermocycler (Stratagene, Cedar Creek, TX) under the following conditions: 1 cycle at 95°C for 10 min, 40 cycles at 95°C for 15 s, 58°C for 1 min, and 72°C for 30 s, and a final cycle at 25°C for 5 s. Unrelated DNA (DNA sample processing control; Luminex, Austin, TX) was added to each specimen prior to extraction to ensure the validity of extraction and DNA amplification. Appropriate positive and negative controls were included with each run.

The reference PCR test conducted at GMF does not include primers or probes specific to *Ehrlichia* spp. or *Rickettsia* spp. Specimens testing positive for these targets by TBP were subjected to nucleic acid sequence analysis (see below) for arbitration of the result. The specimen testing positive for *Rickettsia* spp. at the GMF had been forwarded to the Centers for Disease Control and Prevention (CDC) for confirmation by a *Rickettsia* spp. specific PCR test.

### Nucleic acid sequencing.

Nucleic acid extracts from unresolved samples were evaluated by bidirectional DNA sequencing. A portion of the nucleic acid extract was amplified using Hot Start *Taq* qPCR master mix (New England BioLabs) and “outer” primers based on genomic sequences targeted by TBP and REF PCR tests. Amplification was performed for 35 cycles with initial denaturation for 1 min at 95°C, denaturation for 10 s at 95°C, annealing for 30 s at 50°C (*Borrelia* group 1, *Borrelia* group 2, and *Rickettsia* spp.) and 55°C (*Borrelia* group 1 and Ehrlichia chaffeensis), and extension for 1 min 30 s at 72°C. The PCRs were performed using either the QuantStudio 7 Flex real-time PCR system or the ViiA 7 real-time PCR system (Applied BioSystems). Sample TP280 was amplified using AmpliTaq Gold 360 DNA Polymerase (Applied Biosystems, catalog no. 41398823) with initial denaturation for 10 min at 95°C, followed by 40 cycles of denaturation for 30 s at 95°C, annealing for 30 s at 50°C (*Borrelia* group 1), extension for 1 min at 72°C, and final extension for 7 min at 72°C in a T100 thermal cycler (Bio-Rad). Appropriate amplification products were analyzed by electrophoresis in 2% agarose gel and then forwarded for Sanger DNA sequencing at Retrogen, Inc. (San Diego, CA). Sequencing analysis was done using target-specific “inner primers” and the KB Basecaller algorithm with a Phred Q20 score. Table S1 in the supplemental material lists the positive controls used for amplification/sequencing and the NCBI accession numbers used for alignment.

### Development of synthetic specimens.

Synthetic nucleic acid constructs were developed to assess the ability of the TBP assay to detect each target, including those not encountered in the clinical samples. In addition, specimens containing multiple targets were included to assess the ability of the HDPCR technology to accurately detect and discriminate multiple targets both within in a single fluorometric channel and in different channels. The constructs were chemically synthesized double-stranded DNA fragments designed to be representative of sequences detected by the TBP assay using bioinformatics and strain sequences available from the NCBI. Specifically, sequences present in synthetic constructs are present within the target genes presented in Table 1 and are available in NCBI Gene. Synthetic construct sizes were verified by capillary electrophoresis, and sequences were further confirmed by mass spectrometry (IDT, Coralville, IA). All constructs were diluted to the desired concentration in phosphate-buffered saline (PBS).

A total of 93 synthetic samples that included single and dual target specimens, as well as targets designed to assess species specific exclusivity, were prepared. Specifically, samples containing genetic constructs corresponding to each of the following species were prepared at concentrations of 10^3^, 10^4^, and 10^5^ copies/PCR: B. burgdorferi, *Borrelia* genomospecies 1, *B. californiensis*, *B. mayonii*, *E. chaffeensis*, B. miyamotoi, *R. parkeri*, *R. akari*, *R. philipii*, *R. rickettsii*, *R. felis*, *E. muris* subsp*. eauclarensis*, *B. parkeri*, B. hermsii, *B. turicatae*, B. microti, and *E. ewingii*. An additional 23 specimens were prepared with two different targets present at equal concentrations of either 10^3^ or 10^4^ copies/PCR, including B. burgdorferi*/E. chaffeensis* (10^3^ and 10^4^), B. burgdorferi/B. miyamotoi (10^3^), *Rickettsia* spp*./E. muris* subsp*. eauclarensis* (10^4^), *Rickettsia* spp./A. phagocytophilum (10^4^), *E. muris* subsp. *eauclarensis*/A. phagocytophilum (10^4^), B. hermsii/B. microti (10^4^), B. hermsii*/E. ewingii* (10^4^), B. microti*/E. ewingii* (10^4^), B. burgdorferi*/Rickettsia* spp. (10^3^), B. burgdorferi/B. hermsii (10^3^), *Rickettsia* spp./B. hermsii (10^3^), *E. chaffeensis/E. muris* subsp*. eauclarensis* (10^3^), *E. muris* subsp. *eauclarensis*/B. microti (10^3^), B. miyamotoi/A. phagocytophilum (10^3^), B. miyamotoi/*E. ewingii* (10^3^), A. phagocytophilum/*E. ewingii* (10^3^), B. burgdorferi/A. phagocytophilum (10^3^), B. burgdorferi*/*B. microti (10^3^ and 10^4^), and A. phagocytophilum/B. microti (10^3^ and 10^4^). Finally, a set of 16 specimens that included 10 with no target added and 3 each with *Borrelia bissettii* or Borrelia valaisiana at concentrations of 10^3^, 10^4^, and 10^5^ were included to evaluate specificity and TBP target exclusivity. All specimens were tested in singleton.

### Statistical analysis.

The percent agreement, sensitivity, and specificity and the 95% confidence intervals were calculated using the clinical calculator application available at http://vassarstats.net/clin1.html. Calculation of 95% confidence intervals is based on the method described by Newcombe et al. (19).

## RESULTS

### Study population.

Whole-blood specimens were collected from patients being evaluated for potential tick-borne illness at two medical centers in Wisconsin, Gundersen Health System (GHS) and The Medical College of Wisconsin (MCW). GHS serves a largely rural population in west central Wisconsin with a high endemicity of tick-borne illness. Among the 250 specimens enrolled at GHS, 236 had orders for Lyme serology, 17 (7.2%) of which tested positive. MCW serves a largely urban/suburban population in southeast Wisconsin with a lower endemicity of tick-borne illness. All 175 specimens enrolled at MCW had orders for Lyme serology, 7 (4.0%) of which tested positive. A control group consisting of 40 whole-blood specimens obtained from patients being evaluated for unrelated illnesses at each site were collected to aid in assessment of test specificity. Lyme serostatus in these patients was unknown because testing was not clinically indicated or performed.

### Comparison of HDPCR Tickborne Panel and reference PCR test in patients with suspected tick-borne illness.

A tick-borne pathogen was identified by the HDPCR Tickborne Panel (TBP) or reference PCR (REF) in 27/425 (6.4%) specimens obtained from patients being evaluated for potential tick-borne illness. TBP identified a pathogen in 20/425 (4.7%) of specimens, including A. phagocytophilum (*n* = 11), *Borrelia* group 1 (B. burgdorferi and *B. mayonii*) (*n* = 4), *Borrelia* group 2 (B. hermsii, *B. parkeri*, and *B. turicatae*) (*n* = 1), B. miyamotoi (*n* = 2), *E. chaffeensis* (*n* = 1), and *Rickettsia* spp. (*n* = 2). REF identified a pathogen in 23/425 (5.4%) specimens, including A. phagocytophilum (*n* = 11), *Borrelia* group 1 (B. burgdorferi and *B. mayonii*) (*n* = 9), B. miyamotoi (*n* = 2), and *Rickettsia* spp. (*n* = 1). Notably, 25/250 (10.0%) specimens obtained from GMF were positive by TBP or REF, while only 2/175 (1.1%) specimens obtained from MCW were positive, which is reflective of the difference in endemicity between the two study sites.

TBP and REF results were negative for all 80 blood specimens collected from patients not being evaluated for possible tick-borne illness. In addition, TBP and REF results were in agreement for 17/27 (63%) positive specimens (Table 2). Each test detected A. phagocytophilum (*n* = 11) and B. miyamotoi (*n* = 2) in the same specimens (100% agreement for both targets), and each detected “*Borrelia* group 1” organisms in three other specimens. Among the three specimens testing positive for “*Borrelia* group 1” by both methods, sequence analysis determined one specimen contained *B. mayonii*, while the other two contained B. burgdorferi. Both tests also detected *Rickettsia* spp. in the specimen confirmed by the CDC to contain *R. rickettsii*.

**TABLE 2:** Comparison of HDPCR TBP and reference PCR test in patients with suspected tick-borne illness^*a*^

Target	TBP(+)		TBP(–)		% (95% CI)	
	REF(+)	REF(–)	REF(+)	REF(–)	PPA	NPA
*A. phagocytophilum*	11	0	0	414	100 (68–100)	100 (99–100)
*Borrelia* group 1 (*B. burgdorferi* and *B. mayonii*)	3	1	6	415	33.3 (9–69)	99.8 (98–100)
*Borrelia* group 2 (*B. hermsii*, *B. parkeri*, and *B. turicatae*)	0	1	0	424	ND	99.8 (98–100)
*B. miyamotoi*	2	0	0	423	100 (20–100)	100 (99–100)
*E. chaffeensis*^*b*^	0	1	0	424	ND	NDf
*E. ewingi*^*b*^	0	0	0	425	ND	ND
*E. muris* subsp. *eauclarensis*^*b*^	0	0	0	425	ND	ND
*Rickettsia* spp.	1	1	0	423	100 (5–100)	ND
*B. microti*	0	0	0	425	ND	100 (99–100)

a REF, reference PCR test; PPA, positive percent agreement; NPA, negative percent agreement; ND, not determined.

b The reference PCR (REF) does not contain primers of probes to detect *Ehrlichia* spp. or *Rickettsia* spp. Therefore, PPA and NPA cannot be calculated. Detection of these targets by TBP was compared to sequence analysis or an alternative PCR test that specifically targets these organisms.

A total of 10 discordant results were noted. The TBP identified a pathogen in four specimens (*Rickettsia* spp., *Borrelia* group 1, *Borrelia* group 2, and *E. chaffeensis*) that were negative by REF. Importantly, REF does not contain primers/probes capable of detecting *Rickettsia* spp. or *Ehrlichia* spp., so an accurate assessment of TBP sensitivity for detection of these targets was not possible. However, these specimens were subjected to further analysis, including repeat testing by TBP and nucleic acid sequencing, which did not support the TBP result (see below). REF detected B. burgdorferi in six specimens that were negative by TBP. Of note, all six specimens had threshold cycle (*C_T_*) values ranging from 33.2 to 36.0. In comparison, all specimens testing positive for B. burgdorferi by both TBP and REF yielded lower *C_T_* values (26.5 to 32.9). This suggests a lower sensitivity of TBP for detection of B. burgdorferi in clinical specimens compared to REF, potentially due to differences in the lower limit of detection between the two tests.

### Resolution of discordant results.

Nucleic acid extracts from each of the 10 specimens that generated a discrepant result were retested by both methods and additionally subjected to DNA sequencing. The patients had also been tested for serological evidence of Lyme disease as part of the routine clinical assessment at the time of specimen collection. No specimens with discordant results were serologically positive for Lyme disease at the initial presentation, and results from testing convalescent-phase sera were not available. Therefore, available clinical and additional laboratory information in the medical records were used to aid in adjudication of discrepant results (Table 3).

**TABLE 3:** Resolution of ten discordant results between HDPCR TBP and reference PCR test^*a*^

Specimen	Original result		Repeat result		Sequence analysis	Clinical information	Final determination
	TBP	REF (*C_T_*)	TBP	REF (*C_T_*)			
TBP252	*Rickettsia* spp.	Negative	Negative	ND	Negative	Carpal tunnel pain, paresthesia, fatigue, diaphoresis, and decreased energy	Negative
TBP255	*Borrelia* group 2	Negative	Negative	ND	Negative	Documented tick bite, rash, myalgia, headache, and fatigue	IND
TBP269	*E. chaffeensis*	NA	Negative	NA	Negative	Documented tick bite, myalgia, and “flu-like” symptoms	IND
TBP451	*Borrelia* group 1	Negative	Negative	ND	*B. burgdorferi*	Myalgias, chills, nausea 1 week prior to test	*B. burgdorferi*
TBP265	Negative	*B. burgdorferi* (35.7)	Negative	*B. burgdorferi* (32.8)	Negative	Generalized body aches, EM rash, tick bite, and exposure	*B. burgdorferi*
TBP338	Negative	*B. burgdorferi* (33.3)	Negative	*B. burgdorferi* (31.9)	Negative	*B. burgdorferi* was cultured from PCR-positive blood specimen	*B. burgdorferi*
TBP353	Negative	*B. burgdorferi* (33.2)	*B. burgdorferi*	ND	*B. burgdorferi*	Fever (103°F), exposure, and suspected tick bite	*B. burgdorferi*
TBP408	Negative	*B. burgdorferi* (33.4)	Negative	Negative	Negative	Fever (103°F), body aches, and EM rash; tick bite 7 days prior to test	*B. burgdorferi*
TBP424	Negative	*Borrelia* spp. (36.0)	Negative	Negative	Negative	Arthralgia (multiple joints), myalgia, exposure without obvious bite	IND
TBP469	Negative	*B. burgdorferi* (33.6)	Negative	*B. burgdorferi* (33.3)	Negative	Headache, 3-day history of fatigue, EM rash, and exposure	*B. burgdorferi*

a TBP, HDPCR Tickborne Pathogen Panel; REF, laboratory developed reference PCR test. ND, not determined (insufficient specimen to repeat test); NA, not applicable; IND, indeterminate (insufficient data to make definitive determination of positive or negative; EM, erythema migrans.

Upon repeat testing, the four specimens that originally tested positive only by TBP, including one each for *Rickettsia* spp., *Borrelia* group 1, *Borrelia* group 2, and *E. chaffeensis*, remained negative by REF and also were reported negative by TBP. However, sequence analysis of specimen TBP451 generated a positive result for B. burgdorferi. This patient had a clinical history of chills, myalgia, and nausea for approximately 1 week prior to specimen collection and testing. Based on these data, the specimen was considered a true positive for B. burgdorferi, with a blood concentration near the limit of detection for both the TBP and the REF tests. The patients that tested positive for *Borrelia* group 2 (TBP255) and *E. chaffeensis* (TBP269) also had history of documented tick bite, myalgias, and fatigue, and the *E. chaffeensis* positive patient was treated with a single dose of doxycycline (200 mg) and returned 1 month later with ongoing myalgia, arthralgia, and fatigue. However, repeat TBP and sequence analysis for each of these specimens was negative, so we could not definitively determine whether the original TBP result was accurate. The patient that tested positive for *Rickettsia* spp. by TBP reported carpal tunnel pain, paresthesia, fatigue, diaphoresis, and decreased energy; however, there was no history of tick bite or rash. Repeat TBP and sequence analysis were negative. We therefore considered the original positive TBP finding a false-positive result.

Upon repeat testing of the six specimens that yielded a positive result for B. burgdorferi by only REF, three remained positive with similar *C_T_* values (31.9 to 33.3), but DNA sequencing was negative. Two of the patients (TBP265 and TBP469) had documented erythema migrans (EM) lesions and a history of tick bite and presented with fatigue and myalgia, and B. burgdorferi was recovered by culture from the third patient’s blood sample (TBP338). Based on these data, these three specimens were considered true positives, with organism concentrations below the limit necessary for detection by TBP or DNA sequencing. Of the remaining three discordant specimens, one (TBP353) was not available for repeat REF test but did test positive by TBP upon repeat (originally it had been negative). The patient had a history of fever to 103°F and suspected tick bite. In addition, sequence analysis confirmed the presence of B. burgdorferi in this specimen. Therefore, this specimen was also considered a true positive for B. burgdorferi. The remaining two discordant specimens were negative upon repeat testing by both TBP and REF, as well as DNA sequencing. One of the patients had a history of fever, body aches, EM rash, and documented tick bite 7 days prior to specimen collection and testing (TBP408). Based on these findings, this sample also likely represents a true case of acute infection with B. burgdorferi with a low concentration of organisms (*C_T_* 33.4). The final patient had a history of arthralgia affecting multiple joints, myalgia, and potential exposure to ticks but did not have an obvious bite (TBP424). The original REF *C_T_* value was 36.0, the highest documented value in this study, so we were unable to definitively determine whether the original REF result was a true positive. However, failure of these final two discordant specimens (TBP408 and TBP424) to repeat as positive by REF can be explained by the relatively high *C_T_* values (33.4 and 36.0) obtained in the original result. Evaluation of B. burgdorferi-containing samples at the REF testing laboratory (GMF) confirmed <100% reproducibility of replicate samples when the concentration of spirochetes in the blood was <100 organisms/200 μl (*C_T_* approximately 33). A final comparison of the TBP and REF results based on the data and our conclusions is presented in Table 4.

**TABLE 4:** Comparison of HDPCR TBP and reference PCR based on the composite gold standard^*a*^

Target	No. of samples				% (95% CI)	
	TP	TN	FP	FN	Sensitivity	Specificity
*A. phagocytophilum*						
TBP	11	414	0	0	100 (68–100)	100 (99–100)
REF	11	414	0	0	100 (68–100)	100 (99–100)
						
*Borrelia* group 1 (*B. burgdorferi* and *B. mayonii*)						
TBP	4	416	0	5	44.4 (15–77)	100 (99–100)
REF	8	416	0	1	88.9 (51–99)	100 (99–100)
						
*B. miyamotoi*						
TBP	2	423	0	0	100 (20–100)	100 (99–100)
REF	2	423	0	0	100 (20–100)	100 (99–100)
						
*Rickettsia* spp.						
TBP	1	423	1	0	100 (5–100)	99.8 (98–100)
REF	1	424	0	0	100 (5–100)	100 (99–100)

a Composite gold standard is based on positive or negative agreement between TBP and REF. In cases of discordance, detection of the given target by either sequence analysis or upon repeat testing by either TBP or REF in conjunction with clinical presentation was used to define true positive or true negative. Specimens that could not be resolved based on these additional analyses were considered indeterminant and were not included in this table. See Table 3 for details. TP, true positive; TN, true negative; FP, false positive; FN, false negative.

### Analytic performance using simulated specimens.

A panel of simulated specimens (*n* = 93) was tested to further evaluate the ability of the TBP to identify targets not encountered in the clinical specimens and to assess the ability of the HDPCR methodology to simultaneously detect and identify multiple targets within a single fluorometric channel. The panel contained 54 specimens with a single TBP target at concentrations ranging from 10^3^ to 10^5^ copies/PCR, 26 specimens with multiple targets present at 10^3^ to 10^4^ copies/PCR, and 16 specimens with no target or unrelated targets not expected to be reported by TBP (see Materials and Methods for details). Each simulated specimen was tested by TBP in singleton, and the results are presented in Table 5. Among the 54 specimens that contained a single target, the TBP accurately identified the expected spiked target at each concentration in all samples (100% sensitivity). One false-positive result was observed in sample that contained only the *B. turicate* target but was reported as positive for both *Borrelia* group 2 and A. phagocytophilum. Three additional erroneous results were detected among the 23 specimens that contained multiple targets. This included one incorrect detection of *Borrelia* group 2 in a specimen containing *Rickettsia* spp. and *E. muris* subsp. *eauclarensis*, and two incorrect *Rickettsia* spp. detections in specimens that contained A. phagocytophilum and *E. muris* subsp*. eauclarensis* or B. microti and *E. muris* subsp*. eauclarensis*. In addition, the 16 specimens that did not contain a target or contained unrelated “off-panel” targets (Borrelia valaisiana or *Borrelia bissettii*) were negative.

**TABLE 5:** Performance of HDPCR Tickborne Panel among simulated specimens

Target	No. of samples				% (95% CI)	
	TP	TN	FP	FN	Sensitivity (CI)	Specificity (CI)
*A. phagocytophilum*	10	82	1^*a*^	0	100 (66–100)	98.7 (93–99)
*Borrelia* group 1 (*B. burgdorferi* and *B. mayonii*)	20	73	0	0	100 (80–100)	100 (94–100)
*Borrelia* group 2 (*B. hermsii*, *B. parkeri*, and *B. turicatae*)	13	79	1^*b*^	0	100 (72–100)	98.8 (92–99)
*B. miyamotoi*	7	86	0	0	100 (56–100)	100 (95–100)
*E. chaffeensis*	6	87	0	0	100 (52–100)	100 (95–100)
*E. ewingii*	7	86	0	0	100 (56–100)	100 (95–100)
*E. muris* subsp*. eauclarensis*	7	86	0	0	100 (56–100)	100 (95–100)
*Rickettsia* spp.	19	72	2^*c*^	0	100 (79–100)	97.3 (90–99)
*B. microti*	10	83	0	0	100 (66–100)	100 (94–100)
Total	99	734	4	0	100 (95–100)	99.5 (99–100)

a A. phagocytophilum was called positive in addition to *Borrelia* group 2 in a specimen containing only *B. turicate.*

b *Borrelia* group 2 was called positive in addition to *Rickettsia* spp. and *E. muris* subsp. *eauclarensis* in a specimen containing only *Rickettsia* spp. and *E. muris* subsp. *eauclarensis.*

c *Rickettsia* spp. were called positive in addition to A. phagocytophilum and *E. muris* subsp. *eauclarensis* in one specimen containing only A. phagocytophilum and *E. muris* subsp. *eauclarensis. Rickettsia* spp. were called positive in addition to B. microti and *E. muris* subsp. *eauclarensis* in one specimen containing only B. microti and *E. muris* subsp. *eauclarensis*.

The collective findings therefore showed 100% sensitivity for all single and multiple target specimens across a concentration range of 10^3^ to 10^5^ copies/PCR and >99% (4/738 false positives) specificity, even when the samples contained multiple targets.

## DISCUSSION

Diagnosis of tick-borne infections is complicated by the growing array of pathogens and variable performance of available diagnostic methods. Clinical features such as rash (presence, absence, and appearance), fever, myalgia/arthralgia, hematology (thrombocytopenia, erythrocyte, and leukocyte count) and chemistry (liver enzyme levels) parameters are frequently used to guide test ordering in conjunction with exposure history and geographic distribution of specific tick-borne pathogens (7, 20); however, these clinical features can be nonspecific, and overlap occurs among the various tick-borne pathogens. The expanding geographic ranges of vectors harboring tick-borne pathogens and the identification of novel tick-borne pathogens in these vectors warrants a reassessment of the “norms” for diagnosis of tick-borne diseases (14, 21). This was demonstrated in our own study, which identified a patient with a confirmed positive result for *R. rickettsii*, which is only rarely encountered in west central Wisconsin (7). This patient presented with symptoms of petechial rash, malaise, diarrhea, and vomiting without likely recent travel outside the immediate geographic region. Likewise, our study also identified three patients with B. miyamotoi or *B. mayonii* infections that had negative serologic results for Lyme disease. These likely represent acute infections that would not have been detected based on the laboratory tests ordered by the attending clinicians. Combined, these data support the need for sensitive and more comprehensive testing for patients presenting with generalized symptoms of tick-borne illness.

Molecular diagnostics for tick-borne illness are most valuable during acute infection when serologic tests are frequently negative due to delayed immunologic response or “window” period. Even within this “window,” PCR-based tests for B. burgdorferi are only 12 to 50% sensitive due to the low spirochete load present in blood (frequently <10^3^ genomic copies/ml); however, other species including *B. mayonii*, B. miyamotoi, B. hermsii, and other relapsing fever group *Borrelia* spp. typically reach densities of 10^4^ to 10^6^ spirochetes/ml during acute infection which is more amenable to direct detection using PCR (8, 11, 22). In support, a recent survey using molecular diagnostics to evaluate whole blood specimens collected from patients with suspected tick-borne illness showed that *B. mayonii* and B. miyamotoi were detected more reliably in blood than B. burgdorferi (22). Our findings support this result since the *C_T_* values in the *B. mayonii*- and B. miyamotoi-positive samples were significantly lower than the values detected in the B. burgdorferi-positive samples. In addition to detection of these emerging pathogens, our results also highlighted the clinical utility of testing blood samples from patients with early Lyme disease caused by B. burgdorferi, because none of the nine patients with B. burgdorferi-positive PCR results were serologically positive for Lyme disease at the time of specimen collection. Therefore, the missed diagnoses based on negative serologic results could delay treatment, which in turn increases the potential for dissemination and secondary manifestations, including arthralgia, meningitis, and myocarditis. Moreover, direct detection of the pathogen confirms active infection, while a positive serologic result could indicate current infection or past exposure.

In addition, molecular detection of a specific pathogen obviates the potential for cross-reactivity associated with serologic testing. For example, in this study, a tick-borne pathogen was confirmed in 3/24 (12.5%) specimens with a positive serologic result for Lyme, including two specimens positive for A. phagocytophilum and one positive for B. miyamotoi. In each instance, coinfection was a possibility, but the more likely explanation may have been prior exposure or a lack of specificity of serologic tests. Cross-reactive antibodies that bind B. burgdorferi, B. miyamotoi, and B. hermsii are well documented (13). If this were the case, there would be risk of misdiagnosis of Lyme disease based on the positive serologic result and, while the recommended treatments for Lyme disease, anaplasmosis, and B. miyamotoi infection are identical, differences in the secondary manifestations of each infection and a miscalculated prognosis could impact clinical care (4, 8, 10). Further, there are psychological implications for some patients who receive a diagnosis of Lyme disease, and erroneous reporting contributes to incorrect understanding of the epidemiology of tick-borne infections (23).

Interestingly, we did not identify patients positive for Babesia microti, despite a geographic distribution and vector range nearly identical to that of A. phagocytophilum, B. miyamotoi, *B. mayonii*, and B. burgdorferi. Two explanations could account for these findings. First, up to one-third of B. microti infections in immunocompetent individuals are asymptomatic (24), and the majority of symptomatic infections only cause mild nonspecific symptoms such as fever, headache, and malaise. These patients may not seek medical care or appropriate diagnostic testing, so many infections likely go undiagnosed or unrecognized. Second, while the vector and geographic distribution of B. microti mirrors that of B. burgdorferi and A. phagocytophilum, the incidence of infection is far higher in the northeastern United States (7, 25). Despite these observations, including B. microti on molecular tick-borne pathogen panels remains important because the organism can cause severe illness in immunocompromised individuals, and the treatment differs from that for most other tick-borne pathogens.

A potential drawback of multiplexed diagnostic tests is poor positive predictive value, especially for rarely encountered targets, i.e., even with high test specificity the incidence of a false-positive result exceeds that of true-positive results due to the infrequency of a specific target in the test population. In our study, the TBP identified single specimens as positive for *E. chaffeensis*, *R. rickettsii*, or *Borrelia* group 2, which are each rare in Wisconsin because of the lack of appropriate tick vectors (7). Further, repeat testing and sequence analysis of remaining nucleic acid extracts were negative for these targets. Therefore, we considered these three results as likely falsely positive, though each patient had symptoms consistent with tick-borne infection. To further challenge the specificity of the TBP, we additionally tested 80 blood specimens from patients without clinical test orders suggestive of concern for tick-borne infection. None of these specimens tested positive by either TBP or reference PCR. The collective findings therefore provided strong evidence of high specificity, even when the infection was only rarely encountered. As further support, we observed only four false-positive results among the simulated specimens. Three of the four false-positive results occurred in specimens that contained multiple targets, suggesting the potential for incorrect (additional) detections in complex specimens. Therefore, despite a specificity of >99%, these data reinforce the necessity of correlating laboratory results with clinical presentation, exposure, and travel history, especially in patients with unexpected results.

This study has several limitations that should be considered related to experimental design, as well as the characteristics and performance of the TBP. Among clinical specimens, the TBP demonstrated a sensitivity of only 44.4% (4/9) for the detection of B. burgdorferi compared to a sensitivity of 88.8% (8/9) for the traditional RT-PCR reference test. However, each false-negative result was observed in a specimen with a high *C_T_* value (≥33.2). It is unlikely that freezing and storage of the extracts (up to 4 months) between RT-PCR and TBP testing provides a full explanation for the false-negative results since 3/6 (50%) remained positive when retested by RT-PCR at a similar *C_T_* value (Table 3). A more plausible explanation is a difference in the limit of detection (LoD) between the two assays. The manufacturer claimed the LoD for the TBP assay is 10 to 30 copies/reaction (500 to 1,500 copies/ml), while the LoD of REF is approximately 50 copies/ml for the B. burgdorferi target. Regardless, a negative result should not be used to definitively rule out infection with B. burgdorferi in a patient with compatible symptoms and exposure to ticks.

The TBP reported false-positive results for *Rickettsia* spp. in both clinical (*n* = 1) and simulated specimens (*n* = 2). These errors may be related to the single-channel, multitarget detection HDPCR approach, since the *Rickettsia* spp. target is detected at the lowest fluorescence signal (“level 1”) in channel 1 (Fig. 1), and low-level or background fluorescence may generate a signal that also crosses the signal threshold for this target. This phenomenon could also explain the two false-positive TBP results for the *Borrelia* group 2 target, which is also a “level 1” target in a difference fluorometric channel. Therefore, further optimization of the TBP chemistry or data analysis algorithm may improve the sensitivity for B. burgdorferi and optimize the specificity for “level 1” targets.

The limitations of testing the simulated specimens should also be highlighted. For example, we only tested the synthetic nucleic acid constructs at concentrations that ranged from 10^3^ to 10^5^ copies/PCR. This equates to approximately 5,000 to 500,000 organisms/ml of whole blood, which is not clinically relevant for B. burgdorferi, which rarely exceeds 10^3^ genomic copies/ml during an acute infection. In addition, the synthetic constructs were diluted in PBS rather than in whole blood, which eliminates the potential for inhibition by interfering factors in this matrix. However, Buckwalter et al. showed that inhibition occurred only rarely (<1%) when samples were processed by using nucleic acid extraction procedures similar to those used in this study (26).

It must be noted that the TBP assay and ChromaCode Cloud are designated as “research use only” (RUO), so the manufacturer cannot provide assistance or guidance related to verification or implementation of this assay. Therefore, laboratories that implement the TBP must assume full responsibility for thoroughly establishing performance parameters, including limit of detection, interfering substances, specimen acceptability criteria, precision, accuracy, and reportable and reference ranges. Given the relative infrequency of infections due to some of these TBP targets, it may be difficult to obtain an adequate number of clinical specimens containing each organism to complete a thorough verification study. Characterized reference material, including titered specimens containing whole, intact, heat-inactivated organisms, has recently become commercially available for several of the assay targets and could be considered.

Finally, while the cloud-based approach used in this study offers the advantage of rapid data analysis (approximately 2 min per 96-well plate run), remote access to results, little capital expenditure, and remote IT support. it is possible that data privacy, including unauthorized access to protected health information (PHI), may be a concern. It should be noted that ChromaCode Cloud software is designed to accept only a specimen ID number and does not receive any other associated PHI. Additional risk mitigation is achieved through restricted physical access to servers (hosted by Amazon Web Services [AWS]) and constant monitoring to detect unauthorized electronic access. However, the ChromaCode Cloud analysis program can also be loaded onto a resident computer to provide on-site data analysis and result reporting.

In conclusion, our preliminary clinical evaluation of the HDPCR Tickborne Panel (TBP) using samples spiked with synthetic target yielded high sensitivity and specificity for the simultaneous detection and discrimination of nine pathogens associated with tick-borne infection. More significantly, the findings using clinical samples confirmed high specificity (>98%) and sensitivities of 100.0% for A. phagocytophilum (11/11), B. miyamotoi (2/2), and *Rickettsia* spp. (1/1) and 44% (4/9) for B. burgdorferi compared to a composite gold standard of traditional RT-PCR, sequence analysis, and clinical presentation. A specific benefit of the HDPCR technology is the ability to expand the number of targets identified and differentiated in a single real-time PCR fluorometric channel. This enables compatibility of the TBP assay regents with existing four- or five-channel real-time PCR instruments that are found in most clinical laboratories. The ABI 7500 Fast DX instrument was used for RT-PCR in our study; however, previous studies have demonstrated equivalent performance between ABI PCR systems and the LightCycler 480 system (Roche) (15). Thus, we find the HDPCR Tickborne Panel provides a rapid multiplexed molecular approach to identify nine pathogens or pathogen groups commonly associated with tick-borne illness in the United States and can serve as a viable adjunct for the laboratory diagnosis of tick-borne infections.

## Supplementary Material

Supplemental file 1
