# Supplementary material for: Evaluation of a Novel Multiplex High-Definition PCR Assay for Detection of Tick-Borne Pathogens in Whole-Blood Specimens
Source: J Clin Microbiol. 2019 Oct 23;57(11):e00513-19. doi: 10.1128/JCM.00513-19 (PMC6812998; doi:10.1128/JCM.00513-19)
Supplement: Supplemental file 1 [file JCM.00513-19-s0001.pdf]

Supplemental Table S1. Positive Control and Reference Sequence used for Alignment

HDPCR TBP Assay Species/Grouping	Amplification/Sequencing Positive Control	Reference Sequence (Accession Number NCBI)
Borrelia Group 1	*Borrelia burgdorferi* B31 genomic DNA	*Borrelia burgdorferi* (X16467) and *Borrelia mayonii* (CP015795)
Borrelia Group 2	*Borrelia hermsii* DSM 5251 genomic DNA	*Borrelia hermsii* (BHU65980)
Ehrlichia chaffeensis	*Ehrlichia chaffeensis* NR50090 genomic DNA	*Ehrlichia chaffeensis* (AF474890)
Rickettsia spp.	*Rickettsia rickettsii* NC_009882 gBlock	*Rickettsia parkeri* (KF782322)
